# Supplementary material for: Shift current photovoltaic effect in a ferroelectric charge-transfer complex
Source: Nat Commun. 2017 Aug 17;8:281. doi: 10.1038/s41467-017-00250-y (PMC5561111; doi:10.1038/s41467-017-00250-y)
Supplement: Supplementary file 1 — Supplementary Information [file 41467_2017_250_MOESM1_ESM.pdf]

File name: Supplementary Information
Description: Supplementary figures and supplementary note 1.

File name: Peer Review File
Description:

Supplementary Note 1:

Temperature dependence of photocurrent and photovoltage

In this Supplementary information, we discuss the possible mechanism for temperature dependence of short-circuit photocurrent and open-circuit photovoltage under irradiation of CW light. Although we have not completely understood the temperature dependence of short-circuit photocurrent (Fig. 2c) and open-circuit photovoltage (Fig. 2d), here we first discuss the former on the basis of the data analysis and then discuss the latter with assuming an equivalent circuit.

Supplementary Figure 1 shows the data of short-circuit photocurrent as a function of temperature on a semi-logarithmic scale and their fitting to a thermionic emission model assuming the contact involves a Schottky barrier. The data are well fitted to the formula of

$$J = AT^2 \exp\left(-\frac{e\Phi_B}{kT}\right) \quad (1),$$

where e is the elementary charge, Φ_B the barrier height, k the Boltzmann constant, T temperature, and A the Richardson constant, resulting in Φ_B ~ 40 meV. Considering its narrow bandgap of about 500 meV, the barrier height is within reasonable range. Therefore it is most likely that the short-circuit current is limited by the contact resistance due to a Schottky barrier.

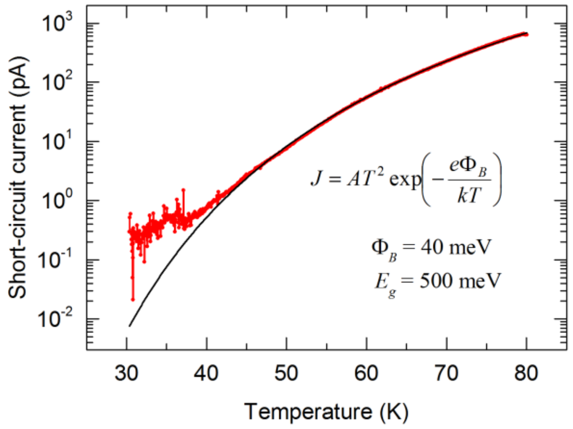

Supplementary Figure 1 | Temperature dependence of short-circuit photocurrent on a semi-logarithmic scale. The black line is a result of the fitting by the thermionic emission model.

Having established a quasi-exponential temperature dependence of contact resistance, we now discuss the temperature dependence of the open-circuit photovoltage with assuming an equivalent circuit model. Supplementary Figure 2 displays the simplest equivalent circuit that may represent device condition during photovoltaic effect measurements, where I_{shift} is shift current which works as a current source, I_{obs} is an observed current, V is a voltage between two electrodes, and R^{*}_{bulk} and R^{*}_{contact} are bulk and contact resistances for photocurrent under illumination, respectively. These parameters are related by the following equation,

$$I_{\mathrm{obs}} = \frac{1}{R^{*}_{\mathrm{bulk}} + R^{*}_{\mathrm{contact}}} V + \frac{R^{*}_{\mathrm{bulk}}}{R^{*}_{\mathrm{bulk}} + R^{*}_{\mathrm{contact}}} I_{\mathrm{shift}} \quad (2).$$

The open-circuit photovoltage (short-circuit photocurrent) is output voltage (current) when I_{obs} = 0 (V = 0). Then, V_{OC} and I_{SC} are given by

$$V_{\mathrm{OC}} = -R^{*}_{\mathrm{bulk}} I_{\mathrm{shift}} \quad (3),$$

$$I_{\mathrm{SC}} = \frac{R^{*}_{\mathrm{bulk}}}{R^{*}_{\mathrm{bulk}} + R^{*}_{\mathrm{contact}}} I_{\mathrm{shift}} \quad (4).$$

Suppose I_{shift} is temperature independent and $R^{*}_{\mathrm{bulk}} \ll R^{*}_{\mathrm{contact}}$, Eq. 4 implies that I_{SC} exponentially decays in low temperature as was shown in Supplementary Fig. 1, whereas Eq. 3 implies that V_{OC} is proportional to R^{*}_{bulk} which can have non-exponential temperature dependence.

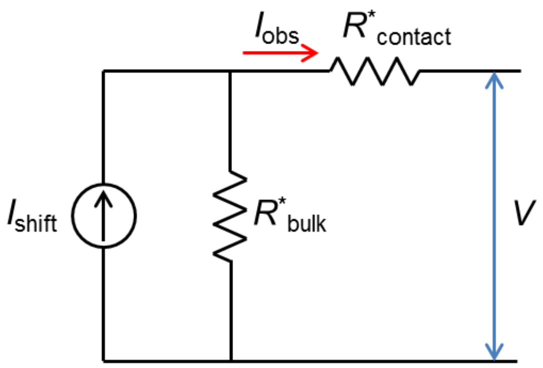

Supplementary Figure 2 | An equivalent circuit for the sample condition during measurements of photovoltaic effect.
